# Repeatability and Reproducibility of Quantitative Corneal Shape Analysis after Orthokeratology Treatment Using Image-Pro Plus Software

**DOI:** 10.1155/2016/1732476

**Published:** 2016-09-28

**Authors:** Ying Mei, Zhiping Tang, Zhouyue Li, Xiao Yang

**Affiliations:** ^1^State Key Laboratory of Ophthalmology, Zhongshan Ophthalmic Center, Sun Yat-sen University, Guangzhou 510060, China; ^2^Tianming Ophthalmology and Optometry Clinic, Kunming, China; ^3^The First Affiliated Hospital of Kunming Medical University, Kunming, China

## Abstract

*Purpose. *To evaluate the repeatability and reproducibility of quantitative analysis of the morphological corneal changes after orthokeratology treatment using “Image-Pro Plus 6.0” software (IPP).* Methods. *Three sets of measurements were obtained: two sets by examiner 1 with 5 days apart and one set by examiner 2 on the same day. Parameters of the eccentric distance, eccentric angle, area, and roundness of the corneal treatment zone were measured using IPP. The intraclass correlation coefficient (ICC) and repetitive coefficient (COR) were used to calculate the repeatability and reproducibility of these three sets of measurements.* Results. *ICC analysis suggested “excellent” reliability of more than 0.885 for all variables, and COR values were less than 10% for all variables within the same examiner. ICC analysis suggested “excellent” reliability for all variables of more than 0.90, and COR values were less than 10% for all variables between different examiners. All extreme values of the eccentric distance and area of the treatment zone pointed to the same material number in three sets of measurements.* Conclusions*. IPP could be used to acquire the exact data of the characteristic morphological corneal changes after orthokeratology treatment with good repeatability and reproducibility. This trial is registered with trial registration number: ChiCTR-IPR-14005505.

## 1. Introduction

Corneal topographers providing qualitative characteristics and quantitative metrics of the anterior corneal surface [1] and transforming raw data into color-coded dioptric power-scale refractive and curvature maps play an important role in orthokeratology fitting. Many recent studies about orthokeratology have mainly investigated the whole corneal morphologic changes by analyzing the following indices: surface asymmetry index (SAI) [2], surface regularity index (SRI) [2], simulated keratometry (SimK) [3], and so forth. Researchers [4, 5] have established that the central corneal epithelium becomes thinner, while the midperipheral cornea becomes thicker. Central corneal flattening corrects axial myopia, whereas midperipheral corneal steepening may act to reduce relative peripheral hyperopia [6, 7]. There is no doubt that the quantitative analysis of the local characteristic morphological corneal changes is much needed after corneal reshaping, such as the changes of the eccentric distance, eccentric angle, area, and roundness. However, it has been challenging to conduct an objectively quantitative analysis of these changes using conventional instruments such as corneal topography software.

Image-Pro Plus software (IPP; produced by Media Cybernetics Corporation, USA) is image analysis software capable of taking information obtained from a photograph and processing it in a variety of ways. In addition, IPP could collect intensity data for entire images or an area of interest, which can offer good results and systematically increasing efficiency. IPP has been used for many biological studies, such as the quantification of proteins [8], formaldehyde [9], and the detection of dyes in food [10] and drinks [11]. IPP has also been applied to eye diseases. Wu et al. [12] used IPP to conduct a precise measurement of the healing area of a wounded cornea. Ye et al. [13] used IPP to investigate the relationship between the pterygium size and ocular residual wavefront aberrations after pterygium surgery.

To the authors' knowledge, image analysis via IPP software has not been used to analyze the corneal shape after orthokeratology treatments, and now it is short of an appropriate method to objectively analyze the local characteristic morphological corneal changes using conventional instruments such as corneal topography software. Therefore, the aim of this study is to evaluate the repeatability and reproducibility of the quantitative analysis of the morphological corneal changes after orthokeratology treatment using the software “Image-Pro Plus 6.0.” It may provide a reference for analysis of corneal reshaping after orthokeratology or corneal refractive surgery.

## 2. Materials and Methods

### 2.1. Materials and Methods

#### 2.1.1. Subjects

Tangential subtractive maps of the topographies from 81 subjects (81 eyes) who were fitted for orthokeratology and wore lenses continuously for more than 20 days in Zhongshan Ophthalmic Center, Sun Yat-sen University, Guangzhou, China, were enrolled. Subjects were given an assent form, and a parent or guardian signed a consent form. The study was conducted in accordance with the tenets of the Declaration of Helsinki and was approved by the hospital's institutional review board (Permit number: 2015QXNL003).

#### 2.1.2. Measurements

Tangential subtractive maps of Medmont E300 (Australia, Medmont Company) before and within 30 ± 10 days after orthokeratology treatment were collected. Sometimes corneal topographic system may produce errors in the recognition of pupillary margin which may cause irregular line of pupil recognition, especially in Asians whose iris is darker. Therefore, before capturing the image, irregular line of pupil recognition needed to be manually adjusted. The step diopter of the tangential subtractive map was set to 0.01 D in custom settings and then captured the whole image with a JPG format setting at a maximum resolution of 1366*∗*768 pixels.

*Exclusion Criteria of the Corneal Topography Maps*.

1. Systematic comprehensive score of corneal topography of less than 95 points.
2. Corneal staining score of ≥3. Corneal staining was graded on a scale of 0 (no staining) to 4 (severe staining) for severity according to the guideline of Mandell's Contact Lens Practice [14].
3. Incomplete bulls eye.

### 2.2. Image Analysis

#### 2.2.1. Definition of Important Parameters

*Treatment Zone*. On the topography map, the central circular zone of corneal flattening after orthokeratology treatment was termed the “treatment zone,” which is surrounded by a ring of midperipheral corneal steepening. The criterion for determining the treatment zone was the region encircled by the inner edge of the “zero diopter change” zone inside the ring of midperipheral steepening on the difference map [15]. To improve the identification precision of the “zero diopter change” zone, the step size of every tangential subtractive map was set to the minimum (0.1 D) in custom settings. In this way, the border between the pseudo colors of +0.05 D and −0.05 D is the boundary of the treatment zone—the “zero diopter change” zone.

*Area*. The area is the one inside the ring of the “zero diopter change” zone.

*Roundness*. Roundness is equal to perimeter^2^/(4^*∗*^ *π* ^*∗*^area) which is the index that reflects the rough shape of the treatment area. The shape became irregular as the roundness was enlarged. When the roundness was close to 1, the treatment area was more round and regular.

*Eccentric Distance*. It is the distance between the center of treatment zone and the pupil center.

*Angle Eccentric*. Angle (0~359°) acquired by counterclockwise rotating around pupil center from 3 o'clock of cornea was defined as decentration angle.

#### 2.2.2. Setting Scale

**Figure 1:**
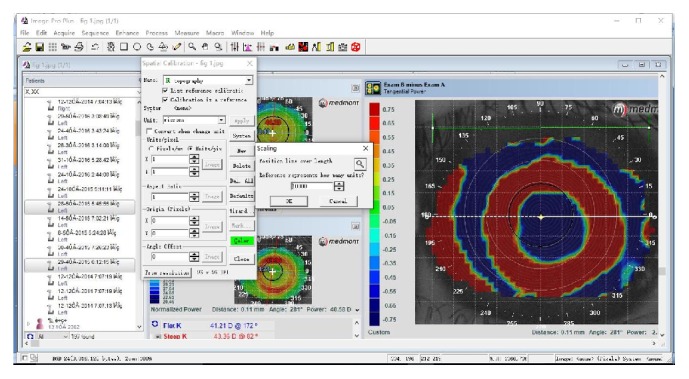
Scale setting.

The measuring scale was set to 1 micron before measurement using IPP software. As shown in Figure 1, we first opened “measure-calibration-spatial” in turn, input “topography” and “microns” as the name and unit, clicked on “image” to show the “position line,” and dragged the “position line” along the “Cartesian grid” of the topographic picture to a length of 10 mm; we then input a “10000” reference to represent 10 mm (Figure 1).

#### 2.2.3. Measurements

After setting the measuring scale, the treatment zone was depicted manually and then the following parameters were measured: the eccentric distance, eccentric angle, area, and roundness of the corneal treatment zone.

Three sets of measurements were conducted for each tangential subtractive map from 81 subjects (81 eyes)—two sets by the same examiner 5 days apart and one set by another examiner on the same day.

### 2.3. Data Analysis

All statistical analyses were performed using SPSS version 18.0 (SPSS 16.0 Inc., Chicago, IL). All data were reported as averages ± standard deviations (SD), and *p* < 0.05 at two tails was considered statistically significant. To assess the repeatability and reproducibility of two different measurements of the first examiner and of the two examiners, the difference, standard deviation, intraclass correlation coefficient (ICC), and coefficient of reproducibility (COR) were calculated in accordance with the method used by Portney and Watkins [16] and Hongmei et al. [17]. ICC > 0.75 was regarded as demonstrating excellent measurement reliability, ICC ≥ 0.4 good reliability, and ICC < 0.4 poor reliability. COR = SD/*n* × 100% (SD: standard deviation of two measurements, *n*: mean value of two measurements).

## 3. Results

### 3.1. General Description of the Measurement Results

Tangential subtractive maps of 81 eyes after orthokeratology treatment were used in this study, including different degrees of decentration, different areas, and morphology of the treatment zone (Table 1). All extreme values of measured parameters pointed to the same material number in three sets of measurements.

### 3.2. Analysis of Repeatability

ICC analysis suggested “excellent” reliability of more than 0.885 for all variables and COR of less than 10% for all variables within the same examiner (Table 2).

### 3.3. Analysis of Reproducibility

ICC analysis suggested “excellent” reliability for all variables of more than 0.90 and COR values of less than 10% for all variables between different examiners (Table 3).

## 4. Discussion

In this study, the eccentric distance, eccentric angle, area, and roundness of the corneal treatment zone acquired by IPP software show good repeatability and reproducibility. Conventional instruments about corneal topography mainly measure the whole corneal morphologic changes, and now it is short of an appropriate method to objectively analyze the local characteristic morphological corneal changes using conventional instruments such as corneal topography software. To our knowledge, this is the first study to propose the method of quantitative analysis by IPP software which is suitable for the analysis of most topography after corneal reshaping with overnight orthokeratology.

**Figure 2:**
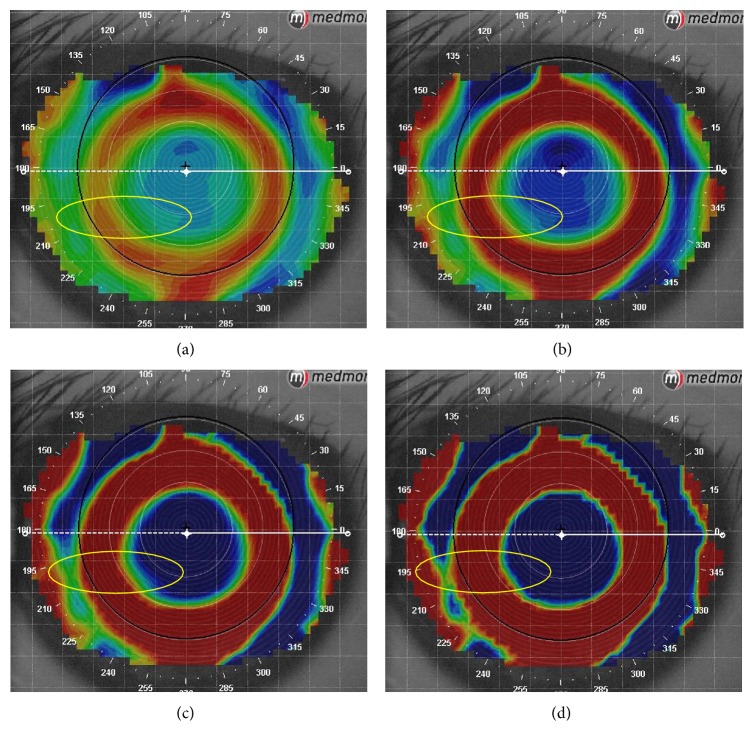
Difference of the same topography read in different “step sizes.” (a) Step size is 1.00 D. (b) Step size is 0.50 D. (c) Step size is 0.25 D. (d) Step size is 0.10 D.

**Figure 3:**
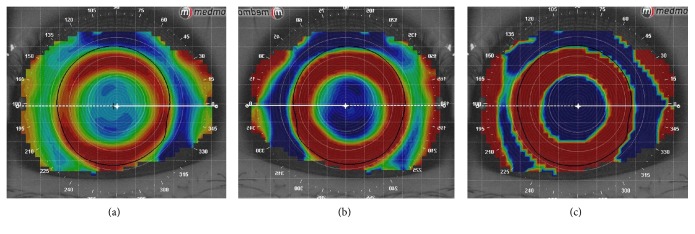
The “inversion arc” became obvious when the “step size” decreased. (a) Step size is 1.00 D. (b) Step size is 0.50 D. (c) Step size is 0.10 D.

According to Sridharan and Swarbrick [15], the central circular zone of corneal flattening after orthokeratology treatment was termed the “treatment zone,” which is from the inner edge to inner edge of the “zero diopter change” zone inside the ring of midperipheral steepening on the difference map. In this study, we found that the “step size” of custom settings had a great effect on the boundary of the treatment zone. As shown in Figure 2, the colorful zone between the “zero diopter change” zones representing the boundary of the treatment zone became wider and less pronounced when the step size of tangential subtractive map was set to the maximum (1.0 D) in custom settings. Conversely, it was narrower and sharper when the step size was set to the minimum (0.1 D). At the same time, when the step size was set to the minimum (0.1 D), the “inversion arc” was the most obvious one, which made it easier to distinguish and depict the treatment zone (Figure 3). Therefore, the step size was set to the minimum (0.1 D) to precisely describe the boundary of the treatment zone in this study.

The treatment zone reflecting the effect of corneal reshaping is important for orthokeratology fitting and corneal refractive surgery, and the eccentric distance and eccentric angle, and so forth, have been used to fix the location of the treatment zone in many clinical studies [15, 18–25]. However, there is still no unified standard reference for depicting the treatment zone, and few studies have investigated the treatment zone using the same method as our study. Since Uozato and Guyton's report [18], surgeons have used the center of the entrance pupil as the center of the optical zone in keratorefractive surgery. Several authors [19–22] have determined the center of laser ablation by putting the cursor on the approximate center of the ablation on the corneal topography maps. Other researchers [23–25] have determined some of the points with the same refractive error by the cursor manually and then found the offset of the treatment zone by the circle or ellipse along with these points. All the methods mentioned above researched the treatment zone as a circle or ellipse. However, the offset of the lens and poor fitting in the clinic would usually cause the treatment zone to be less round or more irregular, and thus it is easy to produce errors when depicting the geometric morphology of the treatment zone in these methods above. In the current study, IPP software could be used to accurately depict the boundary of the treatment zone along with the inner edge of the “zero diopter change” zone manually. After accurately depicting the treatment zone manually, the eccentric distance, eccentric angle, area, and roundness of the corneal treatment zone were analyzed and it showed good repeatability and reproducibility.

In addition to analyzing the indicators mentioned above, IPP software could also be used to analyze many other desired indicators by processing corneal topography images. For example, the pupil size, location, and morphology could be quantitatively analyzed using IPP software. The corneal astigmatism area of topography could be measured to quantify and classify the vertex and peripheral corneal astigmatism. Moreover, some other indicators, such as Box *X*/*Y* (minimum ratio of high to wide of rectangular), Feret (max) (maximum Feret diameter), and Feret (min) (minimum Feret diameter), reflecting the morphological characteristics of the treatment zone could also be measured by IPP software. To our knowledge, this study provided a new idea and an important reference for IPP software to be integrated into the system of topographic software, which may help to acquire and analyze more available parameters by topographic software.

**Figure 4:**
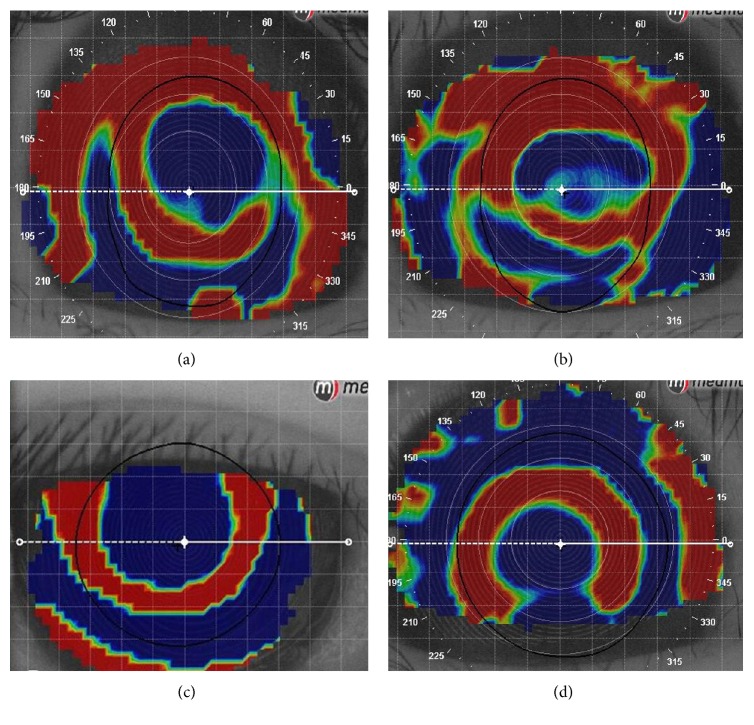
Image of an incomplete “inversion arc.” (a) The incomplete acquisition of image data made the boundary of the “inversion arc” discontinuous. (b) Although the boundary of the “inversion arc” was discontinuous, induced by incomplete acquisition of image data, inner boundary was continuous, so the complete treatment zone could be drawn. (c) The boundary of the “inversion arc” was discontinuous, induced by incomplete acquisition of image data. (d) The boundary of the “inversion arc” was discontinuous, induced by decentration.

In the current study, all topographic images of incomplete bull's eye were excluded which made sure that IPP software could be used to accurately depict the continuous boundary of the treatment zone along with the inner edge of the “zero diopter change” zone manually. Analysis with IPP software was not influenced by any irregular area. Although the roundness of the treatment zone of some topographic images was not so regular, it showed good repeatability and reproducibility. According to the study conducted by Nichols [26], it may influence the stability of corneal topography and the image quality when the corneal staining score was more than 3. In the current study, all the corneal staining scores were less than 2 and systematic comprehensive scores of corneal topography were more than 95 points which confirmed a good image quality recruited in the current study. However, there are several shortcomings of IPP software in the quantitative analysis of topographic maps, including the following. Firstly, the treatment zone could not be drawn completely when the boundary of the “inversion arc” is discontinuous due to the serious offset or incomplete acquisition of image data (Figure 4). It could not be used to analyze massive topographic map images in a timeframe due to its complicated and time-consuming process. Moreover, the boundary of treatment zone needed to be depicted manually which required highly detailed and skilled operation skills. Consequently, it needs a higher demand for examiner which may pose a subjective measurement error. In addition, image processing steps used in the current study are relatively complex and time-consuming which cannot be used for batch processing and analyzing a large number of topographic images. Last, roundness can only roughly depict the morphological characteristics of the treatment zone, but it makes little sense when the morphological characteristics of the treatment zone are seriously irregular.

In conclusion, quantitative analysis of local characteristic morphological corneal changes in the treatment zone after corneal reshaping using the image analysis software IPP had good repeatability and reproducibility, which could be a method for evaluating the effect of corneal reshaping and refractive surgery.

## Figures and Tables

**Table 1:** Measurement results (*n* = 81).

	Eccentric distance (*μ*m)			Eccentric angle (°)			Area of the treatment zone (mm^2^)			Roundness of the treatment zone		
	M1	M2	M3	M1	M2	M3	M1	M2	M3	M1	M2	M3
Mean	561	568	568	206.2	205.8	209.4	9.36	9.19	9.35	1.18	1.19	1.19
SD	262	259	256	65.8	65.5	77.3	2.41	2.58	2.61	0.04	0.04	0.04
Minimum	71	71	81	13.0	17.1	18.4	4.90	4.67	4.87	1.11	1.12	1.12
INMin	81	81	81	80	80	80	23	23	23	80	80	80
Maximum	1340	1324	1346	352.4	352.4	358.6	15.42	15.46	15.56	1.29	1.27	1.32
INMax	42	42	42	61	61	61	31	31	31	52	52	52

M1: the first measurement of the first examiner; M2: the second measurement of the first examiner; M3: measurement of the second examiner; INMin: informational number of the minimum; INMax: informational number of the maximum.

**Table 2:** Repeatability of tangential subtractive maps analyzed by IPP software (*n* = 81).

	M1	M2	M1−M2	ICC	Total mean	COR (%)
Distance (mm)	0.56 ± 0.26	0.57 ± 0.26	−0.01 ± 0.05	0.990	0.56	8.93
Angle (°)	206.24 ± 65.83	205.82 ± 65.51	0.37 ± 3.96	0.999	205.98	1.92
Area (mm^2^)	9.36 ± 2.41	9.19 ± 2.58	0.18 ± 0.12	0.934	9.28	1.29
Roundness	1.18 ± 0.04	1.19 ± 0.04	−0.01 ± 0.03	0.885	1.19	2.52

M1: the first measurement of the first examiner; M2: the second measurement of the first examiner; M1−M2: difference between two measurements before and after the first examiner; ICC: intraclass correlation coefficient; COR: repetitive coefficient; distance: eccentric distance; angle: eccentric angle; area: area of the treatment zone (mm^2^); roundness: roundness of the corneal treatment zone.

**Table 3:** Reproducibility of tangential subtractive maps analyzed by IPP software (*n* = 81).

	M1	M3	M1−M3	ICC	Total mean	COR (%)
Distance (mm)	0.56 ± 0.26	0.57 ± 0.26	−0.01 ± 0.05	0.989	0.56	8.93
Angle (°)	206.24 ± 65.83	209.41 ± 77.35	0.37 ± 3.96	0.946	207.77	1.91
Area (mm^2^)	9.36 ± 2.41	9.35 ± 2.61	0.01 ± 0.88	0.968	9.34	9.42
Roundness	1.18 ± 0.04	1.19 ± 0.04	−0.01 ± 0.02	0.915	1.19	1.68

M1: the first measurement of the first examiner; M2: the second measurement of the first examiner; M1−M3: difference between two measurements before and after the first examiner; ICC: intraclass correlation coefficient; COR: repetitive coefficient; distance: eccentric distance; angle: eccentric angle; area: area of the treatment zone (mm^2^); roundness: roundness of the corneal treatment zone.
